# The role of curvature feedback in the energetics and dynamics of lamprey swimming: A closed-loop model

**DOI:** 10.1371/journal.pcbi.1006324

**Published:** 2018-08-17

**Authors:** Christina L. Hamlet, Kathleen A. Hoffman, Eric D. Tytell, Lisa J. Fauci

**Affiliations:** 1 Department of Mathematics, Bucknell University, Lewisburg, Pennsylvania, United States of America; 2 Department of Mathematics and Statistics, University of Maryland Baltimore County, Baltimore, Maryland, United States of America; 3 Department of Biology, Tufts University, Medford, Massachusetts, United States of America; 4 Department of Mathematics, Tulane University, New Orleans, Louisiana, United States of America; Oxford, UNITED KINGDOM

## Abstract

Like other animals, lampreys have a central pattern generator (CPG) circuit that activates muscles for locomotion and also adjusts the activity to respond to sensory inputs from the environment. Such a feedback system is crucial for responding appropriately to unexpected perturbations, but it is also active during normal unperturbed steady swimming and influences the baseline swimming pattern. In this study, we investigate different functional forms of body curvature-based sensory feedback and evaluate their effects on steady swimming energetics and kinematics, since little is known experimentally about the functional form of curvature feedback. The distributed CPG is modeled as chains of coupled oscillators. Pairs of phase oscillators represent the left and right sides of segments along the lamprey body. These activate muscles that flex the body and move the lamprey through a fluid environment, which is simulated using a full Navier-Stokes model. The emergent curvature of the body then serves as an input to the CPG oscillators, closing the loop. We consider two forms of feedback, each consistent with experimental results on lamprey proprioceptive sensory receptors. The first, referred to as directional feedback, excites or inhibits the oscillators on the same side, depending on the sign of a chosen gain parameter, and has the opposite effect on oscillators on the opposite side. We find that directional feedback does not affect beat frequency, but does change the duration of muscle activity. The second feedback model, referred to as magnitude feedback, provides a symmetric excitatory or inhibitory effect to oscillators on both sides. This model tends to increase beat frequency and reduces the energetic cost to the lamprey when the gain is high and positive. With both types of feedback, the body curvature has a similar magnitude. Thus, these results indicate that the same magnitude of curvature-based feedback on the CPG with different functional forms can cause distinct differences in swimming performance.

## Author summary

When animals move, they receive sensory inputs, which in turn are used to modulate the movement. Relatively little is known about how these inputs affect performance during steady locomotion. Using a computational model of a swimming lamprey, we investigated two different types of feedback, both consistent with experimental data. Both have strong, but different, effects on swimming speed and energy consumption, suggesting that sensory feedback is crucial not just for responding to perturbations, but also for high performance steady locomotion.

## Introduction

To move effectively, all animals must activate their muscles to move their bodies. In nearly every animal studied, this pattern of muscle activation is produced by a relatively small neural circuit called a central pattern generator (CPG) [1, 2], which is influenced by the mechanics of their bodies and the physical world around them. The CPG integrates sensory information, particularly from proprioceptors that sense body movement, and adapts the pattern of muscle activation accordingly [3].

The lamprey has been a model organism for investigating sensorimotor integration during locomotion because its spinal cord, which contains the CPG circuit, can easily be isolated and stimulated to produce a pattern of muscle activity that strongly resembles the pattern in intact swimming animals [4]. Moreover, the primary proprioceptors in lampreys are mechanosensory cells, called edge cells, and are located on the spinal cord [5], in contrast to the proprioceptive muscle spindles and Golgi tendon organs of mammals, which are located in the periphery [6]. These edge cells synapse onto several different classes of ventral horn interneurons that make up the CPG [7]. There are two classes of edge cells: one that inhibits contralateral activity and another that excites ipsilateral activity [7]. Through these connections, edge cells can entrain the CPG to a bending input [5, 8–10] and reset the rhythm after a brief perturbation [11]. In land animals, muscle spindles have similar effects on the CPG [12].

**Fig 1:**
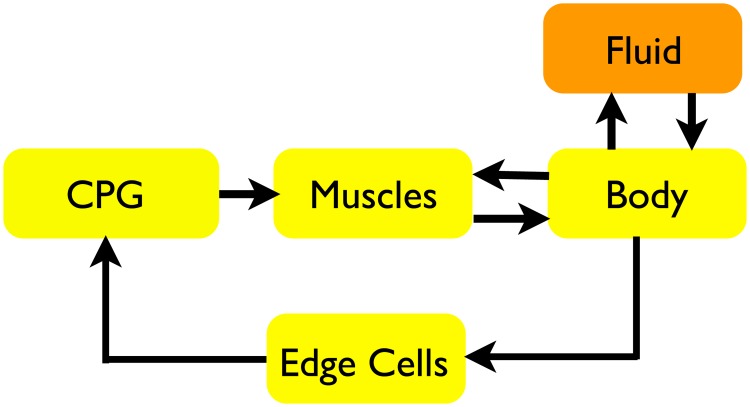
Schematic of feedback loop during locomotion. The CPG generates a signal, driving calcium dynamics that induce muscle contractions. The contractions in turn deform the body which interacts with the surrounding fluid to produce locomotion. Contractile muscle forces also depend upon the length and shortening-velocity of muscle segments. The arrows in the diagram indicate the primary interactions involved in coordinating the different systems to produce swimming behavior. The loop is closed because information on the evolving body curvature impacts the CPG.

Previous studies of edge cells, like those for many other mechanoreceptive sensory cells, have examined their effects in open loop conditions. In these experiments, researchers record the response to different types of mechanical stimuli (*e.g.*, [10, 13]) or they record the effect of edge cells on the CPG due to mechanical inputs (*e.g.*, [8, 9, 11, 14]). However, when a lamprey swims, the edge cells are part of a closed loop system [15]. The CPG activates the muscles, which causes the body to bend, stimulating the edge cells, which then affect the CPG (Fig 1). Edge cells, therefore, not only allow the animal to respond to unexpected perturbations outside the normal pattern of activity, but they can shape the pattern itself. For example, they can cause the rhythm to entrain to a mechanical resonance [16, 17], which may contribute to more efficient swimming.

Here we describe a multiscale computational model of the lamprey closed-loop neuromechanical system. In previous work [16, 18–20], we developed a computational framework that includes a Hill-type muscle model [21] with calcium dynamics [22–24] that drives a passive structural model coupled to a viscous, incompressible Newtonian fluid using an immersed boundary formulation [25–27]. These previous studies examined the effects of varying the stiffness of the body [20], the frequency of activation [20] and the effects of muscle nonlinearities [19]. We found that the same muscle dynamics result in different kinematics when body stiffness is varied, with maximum speed and maximum acceleration occurring at different stiffnesses. Changing the frequency of muscle activation resulted in a change in active lengthening, a phenomenon observed in natural fishes and thought to be related to efficient swimming [20]. Removing non-linear dynamics known to be a part of the muscle force development indicated that without these non-linearities, the same body kinematics can be achieved with very different energy requirements [19]. This previous work focused on the kinematics and effects from changing different aspects of the mechanical parts of the body. Our current study focuses on modeling the central pattern generator and sensory feedback in an effort to understand how information from body mechanics might be communicated to the electrical signal driving the motion.

Other neuromechanical models of undulatory swimming have been developed (*e.g.*, [28, 29]). Iwasaki, Chen and Friesen [30] studied how sensory feedback modulates oscillations in the central pattern generator in leeches. They include models of the CPG, motoneurons, muscle dynamics, sensory feedback and body-fluid interactions, and show robust swimming with adaptivity from sensory input. Both leeches and lamprey exhibit sensory feedback through stretch receptors. Iwasaki et al [30] showed that this feedback in leeches was an essential component in leech swimming. Ekeberg and Grillner [28] used a detailed Hodgkin-Huxley model of neural activation, but a resistive force model, while Gazzola et al. [31] used a fluid model based on elongated body theory with a simple activation wave and proprioceptive feedback modulation. However, because the resistive model of fluid mechanics does not accurately capture the timing or magnitude of forces on the body [20], we chose to incorporate a high-fidelity Navier-Stokes fluid model that can resolve bending dynamics of the lamprey body along with flow features such as the vortex wake shed by the swimmer. Although we are using a two-dimensional fluid model, comparisons with robotic models of anguilliform swimmers and real biological swimmers show excellent agreement in many metrics of flow [18]. The closed-loop model presented here adds a coupled-oscillator representation of the CPG (after [32–34]) with sensory input from stretch receptors that depend on body curvature.

We construct the CPG using chains of nonlinear phase oscillators. Some of the first models to capture key characteristics of experimental studies of the lamprey CPG were phase oscillator models, which define each oscillator based on a single variable, namely its phase [32]. These types of models are simple, yet reproduce some important features of the CPG responses to perturbations [11] along with its entrainment to bending stimuli [8, 33, 35, 36].

Although edge cells have been studied for a long time and their electrical activity is known to be affected by bending, their response properties are only beginning to be characterized. Current experiments indicate that they respond to both the magnitude of bending and the bending rate [13], but their effects on the CPG are not well understood, particularly in a closed-loop system with relatively small deviations from a steady sinusoidal oscillation. Here, we simulate different functional forms of mechanosensory feedback, and investigate how the choice of the feedback model affects swimming performance in the closed-loop neuromechanical system.

Even without feedback, the computational model naturally approximates a subtle feature of fish swimming. When fish are swimming steadily, anterior muscles on one side of the body become active with a small lag after the body is already bending toward that side [37], so that the muscles are always active while shortening and generate positive mechanical power. Although it may sound like this effect is backwards, since muscle force produces curvature, it occurs due to the periodic nature of the oscillation and only appears after several swimming cycles. This phase lag emerges naturally in the computational model [20] and allows the muscles to maintain the ongoing oscillation. Close to the tail, the lag becomes negative, so that muscle activity precedes bending, starting while they are still lengthening under the pressure of fluid forces [37]. This change in phase lag along the body has been suggested to improve the efficiency of fish swimming by stiffening the tail so that anterior muscle power is more effectively transmitted to the fluid [38, 39]. To quantify the lag, we compare the relative wave speeds of the mechanical curvature wave and the neural activation wave. When the curvature wave is slower than the neural activation wave, the posterior muscle ends up being active during lengthening (see, e.g., [23]), as is seen in fishes [37].

We examined two classes of feedback based on body curvature. In the first, which we term directional feedback, the CPG receives an input that contains the magnitude and direction of curvature. We find that this feedback alters the relative timing of left and right side activity, changing the duration of activity without altering the overall frequency. This type of effect is similar to the well-known phasic effects of edge cells in lampreys [11, 40] and of muscle afferents in cats and other tetrapods [3, 12]. In the second class of feedback, termed magnitude feedback, the CPG receives an input that only contains the magnitude of the curvature, not its direction. Depending on the sign of the feedback gain, we find that this type of feedback is either generally excitatory or inhibitory, speeding up or slowing down the overall oscillation without altering the left-right phasing. This feedback produces an effect that is similar to the excitatory effect of edge cells in the lamprey [41, 42], and of proprioceptive input on vertebrate CPGs in general [3].

In summary, we demonstrate that proprioceptive feedback to the CPG that uses both magnitude and direction of body curvature alters the duty cycle of muscle activation, whereas proprioceptive feedback that uses only the magnitude of body curvature alters the frequency of the activation cycle. Below we will show that these two effects, in the full integrated model, alter swimming kinematics and energetics in complex ways. These results show that the model CPG is capable of generating very different responses depending on whether or not the proprioceptive feedback provides directional information.

## Methods

In this section, we describe each model component (Fig 1) (CPG, muscle, body, fluid and sensory edge cells) and indicate how these subsystems interact in a way that leads to swimming.

### Body structure

**Fig 2:**
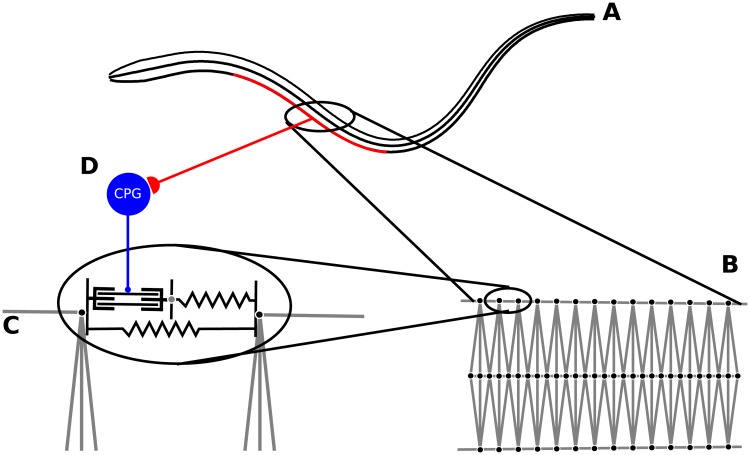
Schematic of lamprey body structure. **A:** The body of the computational lamprey is comprised of three filaments. **B:** This zoomed-in portion of the body indicates the spring-connections between discrete nodes of the filaments that will generate passive elastic forces. Springs connecting nodes along the centerline to other nodes are standard Hookean springs. **C:** Force generation due to muscle segments connecting discrete nodes along lateral sides will evolve using a Hill-type model. The oscillator below the spring-mass damper system is a spring representing the skin of the animal, resisting extension beyond the rest length, but not compression shorter than the rest length. **D:** A single oscillator, from the double chain of oscillators that represent the lamprey CPG, will determine the activation of its corresponding muscle segment.

As illustrated in Fig 2, the body model of the lamprey includes components that support both passive forces that represent the mechanics of the body and active forces due to contractile muscle segments that bend the body to enable swimming. The signal from the CPG activates the muscles segments. The passive and active forces along the lamprey are coupled to the surrounding viscous, incompressible fluid. Fig 2A shows that the body is comprised of three filaments, one for each lateral side **X**_*j*_(*s*, *t*), *j* = 1, 2 and one representing the midline **X**_3_(*s*, *t*). In all simulations presented, the length of the midline of the lamprey was chosen to be *L* = 12.56 cm in the average range of lamprey lengths used in experiments [22].

There are two forms of tapering in our model. First, the body becomes narrower toward the tail (Fig in S2 Fig) Second, we account for the decrease in the physiological cross-sectional area of muscle in the posterior body. Total muscle force is proportional to its cross sectional area. Therefore, we scale muscle force accordingly in the tapered region, assuming that the 2D representation of the lamprey has elliptical cross-sections out of the plane.

The first eighth of the body represents the passive head region with no muscular activity, while waves of muscular contraction can act on the rest of its length to propel the animal through the fluid. Fig 2B shows an enlarged portion of the body, and illustrates the discretization of the segmented filaments that will be used in an immersed boundary framework. The stiff midline filament is discretized into 640 segments and the two lateral filaments are discretized into 320 segments each. Connections between the nodes along the midline and the crosslinks that connect the midline to the lateral sides are modeled as passive Hookean springs. The use of Hookean springs rather than damped springs is possible without the danger of lateral oscillations due to the inherent damping effects of the surrounding viscous fluid. The links along the lateral sides, enlarged in Fig 2C, represent a model of the muscle and the skin. The muscle model supports active muscle contractions and is based upon the kinetic model presented in the section “Muscle Model” below. The skin is modeled using springs that only resist extension, but not compression, similar to collagen fibers (bottom spring in Fig 2C). Given the individual stiffness constants of the structural springs comprising the discretized lamprey body, its overall average macroscopic bending stiffness *E* can be computed [20, 43]. This structural model was used in [20] to explore the role of body stiffness in the swimming performance of the lamprey for a prescribed wave of muscle activation (i.e. no CPG). Note that for the simulations presented in this manuscript, we use a fixed bending rigidity (*E* = 0.76 MPa) throughout.

### CPG

Muscles are activated by an electrical signal from the CPG. Our previous model of lamprey swimming prescribed a wave of neural activation to simulate the input of the CPG, but this neural activation was not informed by the evolving dynamics [20].

In contrast, here we simulate the full Navier-Stokes fluid dynamics, where fluid effects on evolving body curvature are captured, along with the dynamics of the vortex shedding in the wake of the tail movement. Although we are using a two-dimensional fluid model, comparisons with robotic models of anguilliform swimmers and real biological swimmers show excellent agreement in many flow metrics [18].

**Fig 3:**
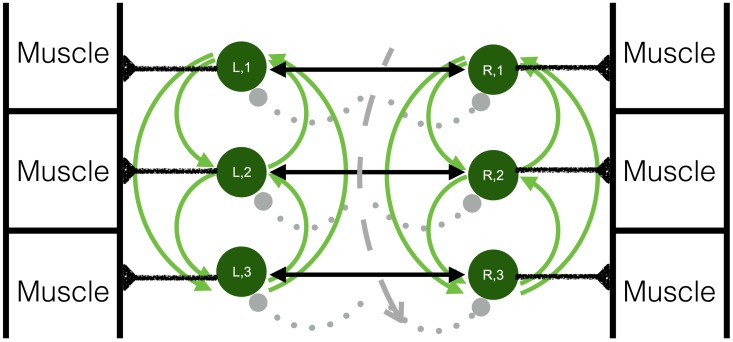
Coupling of the oscillators. To construct the phase oscillator model of the CPG, each oscillator is connected to every other oscillator on the same lateral (“L = left” or “R = right”) side (solid green lines). Within one segment, numbered from head to tail, the left and right oscillators are also coupled together (solid black lines). The dotted gray lines represent curvature and its influence on the CPG oscillators. The curvature at each segment is calculated such that positive curvature curves in the direction of the right side measured from head to tail.

The CPG is modeled using a double chain of sinusoidally coupled phase oscillators (one for the left lateral side and one for the right, see Fig 3):

$$\begin{array}{c} \frac{{d\theta }_{k,i}}{dt}=\omega +\sum_{j=1}^{n}\alpha_{i,j}\sin(\theta_{k,j}-\theta_{k,i}-\psi_{ij})+\alpha_{c}\sin(\theta_{k,i}-\theta_{k*,i}+\pi )+\eta (\kappa_{i}). \end{array}$$ (1)

Here, *θ*_*k*,*i*_ represents the phase of the *i*^*th*^ oscillator in the chain, on the *k*^*th*^ side, where *k* = 1 represents the right side, and *k* = 2 represents the left side. The notation k* indicates the opposite side, in other words, if *k* = 1, then k* = 2. The natural frequency of these oscillators is denoted by *ω* in Eq (1), and throughout this work we choose *ω* = 2*π* rad/sec.

The *i*^*th*^ oscillator on the *k*^*th*^ side is coupled to the other oscillators on the same side with strength given by

$$\begin{array}{c} \alpha_{i,j}=\{\begin{array}{cc} A_{a}\exp(-\frac{\left|i-j\right|}{\lambda_{a}}) & i-j<0\\ A_{d}\exp(-\frac{\left|i-j\right|}{\lambda_{d}}) & i-j>0, \end{array} \end{array}$$ (2)

where |*i* − *j*| is the number of segments between between oscillator *i* and oscillator *j*. As in [36], we choose the parameters *A*_*a*_ = 1.0 rad/sec, *A*_*d*_ = 10 rad/sec, λ_*d*_ = 5, and λ_*a*_ = 40. The asymmetry in the coupling strengths *A*_*a*_ and *A*_*d*_ is consistent with experimental data [36, 44]. We tune the system with a constant phase shift between neighboring oscillators, so that $\psi_{ij}=\left(i-j\right)\bar{\psi }$. Because in these simulations there are 280 segments on each lateral side that comprise the active portion of the lamprey body, we choose $\bar{\psi }=\frac{2\pi }{280}$, which corresponds to the natural phase shifts found experimentally in [40, 45].

The term *α*_*c*_ sin(*θ*_*k*,*i*_ − *θ*_k*,*i*_) in Eq (1) represents the observed antiphase behavior between left and right segments which couples oscillator *i* on the *k*^*th*^ side to oscillator *i* on the opposite side. The coupling strength *α*_*c*_ = 81.87 *rad*/*sec* was chosen to be ten times the strongest descending coupling connection. We choose the connection across a segment to be stronger than the lateral connections because it has been observed that activation waves travel in anti-phase, and that co-contraction is not favored in undulatory swimming [36].

Finally, the term *η*(*κ*_*i*_) in Eq (1) captures proprioceptive feedback to the CPG system. Here *κ*_*i*_ is the curvature of the lamprey midline at segment *i*, and the choice of the functional form of *η*, the feedback, will be explored below.

### Muscle model

The signal that the CPG sends to activate the muscle segment is modeled based on the phase of the oscillator on that side. If the muscle segment is sent an activation signal by the CPG, free calcium in that muscle segment is taken up by the thick filaments, generating contractile force. As in Williams [22], we use a mass-action kinetic model of calcium dynamics in each muscle segment and couple it to a Hill-type model of muscle force generation, modified based upon data from lamprey experiments. In Hamlet et al. [19], we explored the implications of muscle nonlinearities on the swimming performance of the lamprey model (with a prescribed activation wave taken as input) by varying the dependence of muscle force generation on segment length and shortening velocities. Here, however, we choose the same parameters in the muscle model in each simulation. We briefly describe the elements of muscle force generation as in Hamlet et al. [19].

#### Calcium dynamics

**Fig 4:**
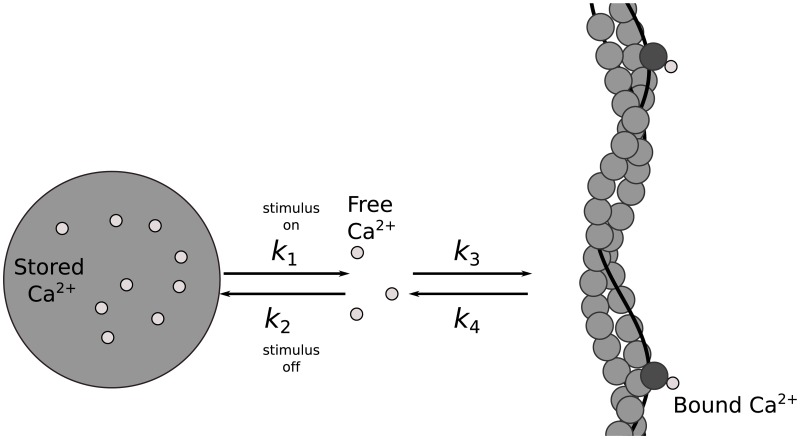
Fig showing calcium dynamics (as seen in [22]) during an active contraction cycle. Free calcium ions (*C*_*f*_) are stored in the sarcoplasmic reticulum (SR) until the muscle is activated. During activation, calcium ions are released from the SR and bind to troponin (bound calcium denoted *C*_*b*_) along the actin filament, inducing contractions. The values *k*_*n*_ and the arrows indicate the rate and equilibrium dynamics of each reaction, respectively.

Calcium is required to activate the contractile elements in muscle [21]. Here we adopt the model of Williams [22] that tracks both free calcium ions and calcium ions bound to a thick filament, and includes length dependence, velocity dependence, and work-dependent deactivation (see Fig 4). The model assumes that the sarcoplasmic reticulum will release free calcium ions, based on a signal from the CPG. Once the calcium ions are released, they can bind to myofibrils and induce contraction of the muscle. The reaction is reversible, in the sense that calcium ions may unbind from the myofibrils and resequester in the sarcoplasmic reticulum. The amount of calcium ions bound to myofibrils will impact the force generation of the contractile element in the Hill-type model described below.

**Fig 5:**
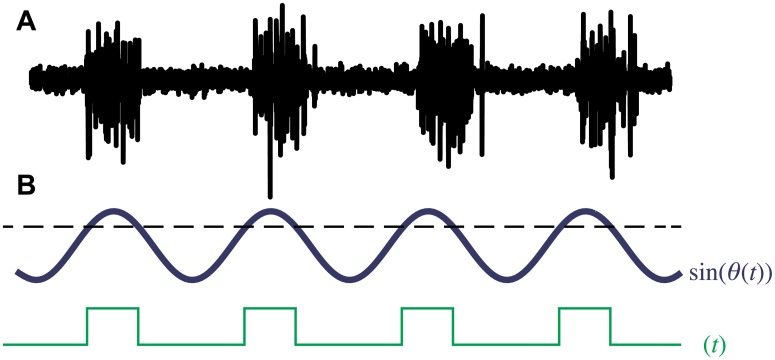
Construction of an activation wave from the CPG model. **A:** Bursting pattern from a typical CPG neuron in the lamprey (after [32]). **B:** Generation of the activation signal *σ*(*t*) based on thresholding the CPG output *θ*(*t*).

Muscle is innervated when a signal from the CPG reaches a pre-defined threshold. Fig 5 illustrates how the phase oscillator model is used to capture the experimental bursting pattern. Fig 5A shows a typical bursting pattern from an oscillator in the CPG and Fig 5B shows result of taking the sine of the phase of a model oscillator of the CPG. Note that the frequency of the bursting here corresponds to the periodicity of the sinusoidal function. Given a phase *θ*(*t*), we define the time interval that corresponds to the segment being “active” to be those times when sin *θ*(*t*) is above a prescribed threshold *τ*. Fig 5B shows how an “on-off” activation signal *σ*(*t*) may be extracted from the phase *θ*(*t*). For the phases along the segments on the lateral sides of the lamprey model, we then have

$$\begin{array}{c} \sigma_{ik}\left(t\right)=\{\begin{array}{cc} 0 & \sin(\theta_{ik}\left(t\right))<\tau \\ 1 & \sin(\theta_{ik}\left(t\right))\geq \tau . \end{array} \end{array}$$ (3)

The CPG signals a release of calcium ions from the sarcoplasmic reticulum, which is then available for binding on the myofibrils. Let *C*_*f*_ denote the free calcium ions released from the sarcoplasmic reticulum and *C*_*b*_ be the amount of calcium ions bound to the myofibrils. For each oscillator/muscle unit, the dynamics of these chemical populations are governed by

$$\begin{array}{c} \frac{dC_{f}}{dt}=(k_{4}C_{b}-k_{3}C_{f})(1-C_{b})+k_{1}\left(C-C_{f}-C_{b}\right)\\ \;+k_{2}(C_{f}(C-S-C_{f}-C_{b})) \end{array}$$ (4)

$$\begin{array}{c} \frac{dC_{b}}{dt}=-(k_{4}C_{b}-k_{3}C_{f})(1-C_{b}), \end{array}$$ (5)

where *S* is the number of sequestering sites present in the sarcoplasmic reticulum, *C* is the total amount of calcium ions, free or bound, in the entire system, and *k*_1_ − *k*_4_ are rate constants for the binding and unbinding of calcium [23]. The values of *k*_1_, *k*_2_, *k*_3_, *k*_4_ at the *i*, *k*th segment in Eqs (5) and (4) are given by

$$\begin{array}{c} k_{1}=\sigma_{ik}{\bar{k}}_{1} \end{array}$$ (6)

$$\begin{array}{c} k_{2}=\left(1-\sigma_{ik}\right){\bar{k}}_{2} \end{array}$$ (7)

$$\begin{array}{c} k_{3}=\frac{{\bar{k}}_{3}}{\sqrt{m}} \end{array}$$ (8)

$$\begin{array}{c} k_{4}={\bar{k}}_{4}\sqrt{m} \end{array}$$ (9)

where ${\bar{k}}_{1},{\bar{k}}_{2},{\bar{k}}_{3},{\bar{k}}_{4}$ are all baseline constants that were fitted to match experimental data [19]. The values of *C*_*f*_, *C*_*b*_, *C*, and *S* have been nondimensionalized against the number of available binding sites and are themselves taken to be unitless. The relative proportions are such that the total number of sequester sites, *S* is greater than *C* and that the total amount of calcium, *C* is greater than the number of available binding sites, ensuring that the muscle may be fully activated and the calcium may be fully sequestered during the cycle. The rate constants were then fitted to match experimental results shown in [22]. Note that signal from the CPG feeds into the calcium model here through the values of *k*_1_ and *k*_2_. The parameters *k*_3_ and *k*_4_ correspond to the free calcium binding and unbinding to the myofibrils and depend on *m*, which measures how much work the muscles have done during the current cycle. The variable *m* follows the dynamics

$$\begin{array}{c} \frac{dm}{dt}=\{\begin{array}{cc} -k_{m1}P_{c}v_{c} & v_{c}<0\\ -k_{m2}(m-1) & v_{c}\geq 0 \end{array}, \end{array}$$ (10)

where *k*_*m*1_ and *k*_*m*2_ are parameters from [22] fitted to data, and *P*_*c*_ and *v*_*c*_ are the force and the velocity of the contracting part of the muscle segment (discussed in the next section). This model of work-dependent deactivation hypothesizes that the amount of work a muscle unit can do depends on how much work it has already done as well as how quickly the force developed by a muscle decays once activation has ceased [19, 22].

### Force generation

Each muscle segment is modeled as a modified Hill-type muscle model that consists of a contractile element (CE), that develops force by contracting in response to an activation signal, and an elastic stretch element (SE), that stores and releases energy from force developed by the contractile element. Here, we assume the contractile force depends upon both length and velocity of the stretch, as well as the amount of bound calcium in the system. The stretch element is described by a spring-mass-damper system driven by the contractile force. To account for tapering along the body, the magnitude of the force is scaled by the ratio of the cross-sectional area of the segment and the maximum cross-sectional area of the body.

The contractile force has the form

$$\begin{array}{c} P_{c}=P_{0}\alpha \left(v_{c}\right)\lambda \left(l_{c}\right)C_{b}, \end{array}$$ (11)

where *α* is a nonlinear function of *v*_*c*_, the velocity of shortening of the CE; λ is a nonlinear function of *l*_*c*_, the length of the CE; *C*_*b*_ is the bound calcium given by Eq (5); and *P*_0_ is a constant scale factor indicating the maximal contractile force. For more details on this muscle model, we refer the reader to [19]. In particular, all simulations described below assume work-dependent de-activation and calcium-dependent passive stiffness of muscle elements, corresponding to the case *VLWσ* in [19].

### Sensory feedback

The CPG responds to sensory feedback. Here we model proprioceptive (body-sensing) feedback from edge cells. As discussed above, edge cells are mechanoreceptors [2, 5] that sense stretch along the body and send signals that serve to inhibit the contralateral side of the body and excite the ipsilateral side (relative to the edge cell’s position) with increasing stretch. Recent results of Massarelli et al. [33] have shown that edge cells also respond to rate of change of stretch. Here, for simplicity, we restrict our feedback model to depend only on stretch, which is done by monitoring body curvature. Details regarding the functional form of the input from the edge cells to CPG are not known at this time. To represent the feedback from edge cells to the CPG, we add a feedback term in Eq (1) of the form *η*(*κ*_*i*_) where *κ*_*i*_ is the curvature of the lamprey midline at segment *i*. As a starting point, we model this as an additive response. At each time step, curvature is calculated along the centerline of the body from head to tail at a point (*x*(*s*), *y*(*s*)) by

$$\begin{array}{c} \kappa =\frac{x^{\prime }y^{\prime \prime }-y^{\prime }x^{\prime \prime }}{{\left({\left(x^{\prime }\right)}^{2}+{\left(y^{\prime }\right)}^{2}\right)}^{3/2}} \end{array}$$ (12)

where ′ denotes derivative with respect to the spatial parameterization *s*, *x* is the longitudinal direction and *y* is the transverse direction. In practice, these spatial derivatives are replaced by finite differences, and we smooth this curvature at segment *i* by replacing it by the average curvature of the neighboring segments from *i* − 5 to *i* + 5. The last 5 segments of the body receive no input from the curvature of the body. The value of curvature varies both along the lamprey body and in time as the body bends into an S-shaped swimming pattern. Positive curvature represents bending to the right. When curvature magnitude is high, one side of the body is stretched and the other is compressed.

We chose to compare two different functional forms of the feedback:

$$\begin{array}{c} \eta \left(\kappa_{i}\right)=\eta_{m}|\kappa_{i}|, \end{array}$$ (13)

which we call magnitude feedback (denoted M), and

$$\begin{array}{c} \eta \left(\kappa_{i}\right)={\left(-1\right)}^{k}\eta_{d}\kappa_{i}, \end{array}$$ (14)

which we call directional feedback (denoted D), and where *i* is the segment number, *k* is the side of the body (1 is left and 2 is right), and *η*_*m*_ and *η*_*d*_ are the constant gain parameters with units of cm rad/sec. We will discuss the implications of these feedback forms in the Results section below.

### Fluid-structure interactions

The neutrally-buoyant lamprey body supports passive forces due to its elastic linkages as well as active forces along the lateral sides due to muscle contractions. The strength and timing of the contractions of individual muscle segments evolve with the CPG, the calcium dynamics, and the Hill-type muscle model that are each coupled to the evolving body shape. We adopt an immersed boundary framework [27] that couples the forces supported along the three filaments of the lamprey body, together denoted by **X**(*s*, *t*), to a surrounding viscous, incompressible fluid:

$$\begin{array}{c} \rho [\frac{\partial u(x,t)}{\partial t}+u(x,t)\cdot \nabla u(x,t)]=-\nabla p(x,t)+\mu \nabla^{2}u(x,t)+f(x,t) \end{array}$$ (15)

$$\begin{array}{c} \nabla \cdot u(x,t)=0 \end{array}$$ (16)

$$\begin{array}{c} f(x,t)=\int_{\Gamma }F(X(s,t),t)\delta (x-X(s,t))ds \end{array}$$ (17)

$$\begin{array}{c} \frac{\partial X}{\partial t}=u(X(s,t),t)=\int_{\Omega }u(x,t)\delta (x-X(s,t))dx. \end{array}$$ (18)

Eqs (15) and (16) are the incompressible Navier-Stokes equations, where **u** is the fluid velocity field, *p* is the pressure, *ρ* = 1 g · cm^−3^ is the fluid density, *μ* = 1 mPa · s is the fluid viscosity, and *δ* is the two-dimensional Dirac delta function. Eq (17) communicates the Lagrangian forces **F**(**X**(*s*, *t*), *t*) supported on the lamprey (Γ) to Eulerian forces defined at any point **x** in the fluid domain (Ω). Eq (18) enforces the no-slip condition that says the velocity of a material point of the lamprey body is equal to the fluid velocity evaluated at that point. Note that the forces **F**(**X**(*s*, *t*), *t*) contain contributions due to the deformation of the passive elastic structure as well as actuated muscle contractions.

The immersed boundary formulation in Eqs (15), (16), (17) and (18) uses a continuum description of both the fluid domain and the immersed lamprey body. In practice, the filaments of the lamprey are represented by discrete nodes, and the fluid equations are discretized on adaptive finite-difference grids. To communicate forces and velocities between the Eulerian fluid grid and the Lagrangian nodes of the lamprey, a grid-dependent, regularized approximation of the Dirac delta function is used [25, 27]. Because both the passive elastic linkages and the contractile muscle segments produce equal and opposite forces at their endpoints, the neutrally-buoyant lamprey is a free-swimmer that generates zero total force and torque at each instant of time. We interface our structural model with the parallelized, adaptive mesh implementation of the immersed boundary method (IBAMR) (developed by Griffith [25]) that allows us to achieve the spatial resolution necessary to resolve boundary layers at physiologically-appropriate Reynolds numbers. The adaptive mesh refinement uses a finer fluid mesh near the nodes of the lamprey as well as in fluid regions where a vorticity exceeds a specified threshold. The fluid domain was defined to be 7.5 body lengths long and 3.0 body lengths high. In the simulations presented here, we allowed five levels of refinement, with the coarsest discretization a 32 cell grid in the *x*-direction over the entire domain, and the finest level effectively a 512 cell grid in the *x*-direction. No-stress boundary conditions on the computational domain were implemented. The simulations were run for a total of 10 seconds of simulated time which was determined to be a sufficient amount of time for each case to achieve and maintain a steady swimming speed. Each simulation was run on a linux cluster comprised of 8-core 2.4-2.8GHz AMD Operon processors with 128GB of RAM per core. The simulations were each run on 8 processors on a single node and ran on the order of a day to complete 10 seconds of simulated time. Variation in runtimes was due primarily to differences in the evolving adaptive mesh refinements in the Navier-Stokes solver. Initially, the fluid velocity was at rest and the lamprey body was in a horizontal position, with all elastic linkages at their rest lengths. Convergence studies of this lamprey-fluid immersed boundary model were presented in [18].

### Phase lag

**Fig 6:**
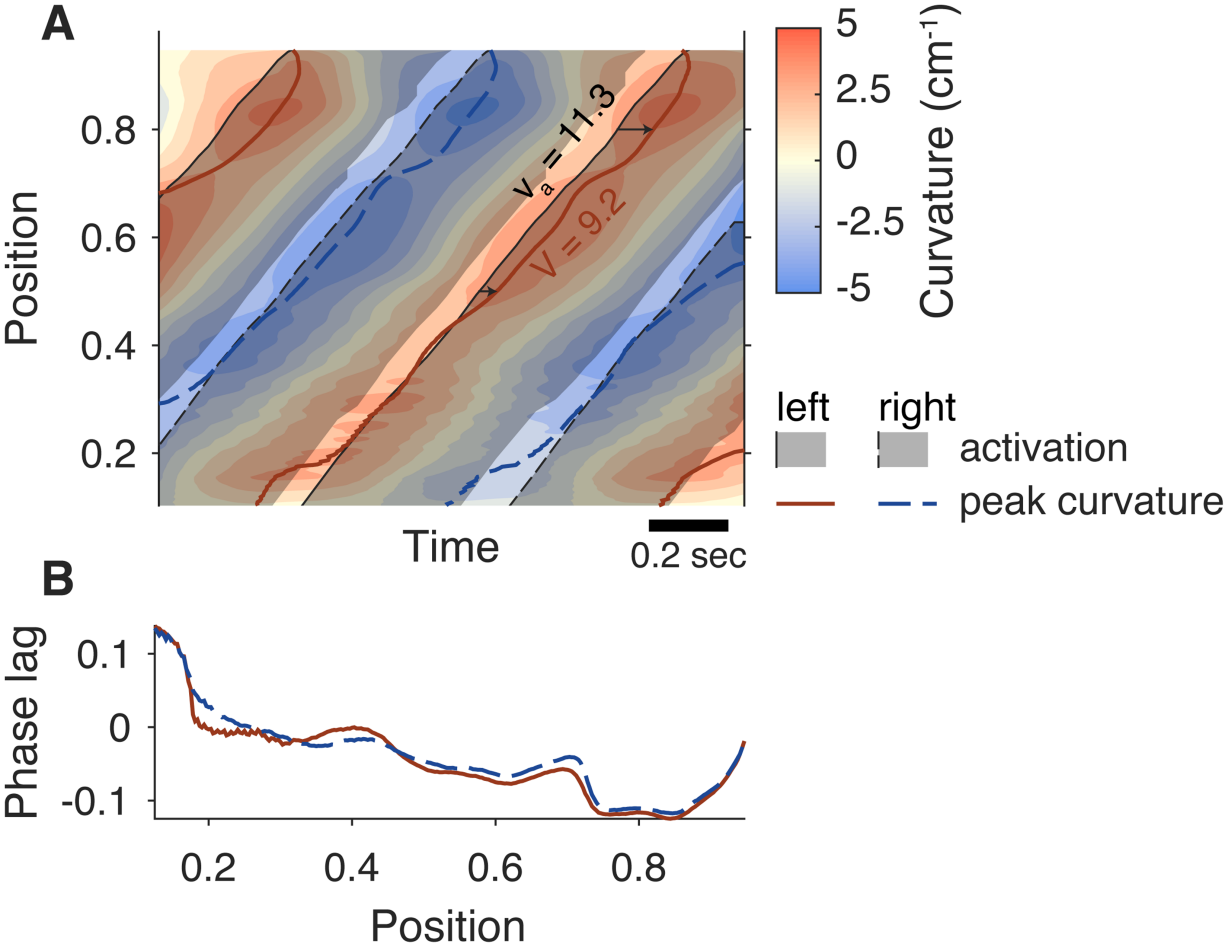
Schematic showing the definition of phase lag and activation and curvature wave speeds. **A:** Curvature (red and blue) and activation (gray shaded regions) as a function of position on the body and time. Phase lag is defined as the time between peak curvature (red and blue lines) and the onset of activation (solid and dashed black lines), indicated by small black arrows. Curvature wave speed *V* is the average slope of the position of peak curvature with respect to time and activation wave speed *v*_*a*_ is the slope of the body position where activation starts, with respect to time. **B:** Phase lag with respect to position for the left side (red) and right side (blue). Note that it becomes negative and larger in magnitude at more posterior positions.

Because of the coupling between the body and the fluid, the simulation naturally develops a phase lag in which muscle activation comes after body curvature, even without feedback [20]. This effect is due to the periodic nature of the swimming oscillation and stabilizes several swimming cycles after the simulation starts. For the current model, we computed the phase lag between muscle activation and curvature, as shown in Fig 6. We identified the time of peaks in curvature ($t_{curve,i}^{L}$, $t_{curve,i}^{R}$) and the closest muscle activation time ($t_{act,i}^{L}$, $t_{act,i}^{R}$) (Fig 6A) for each point along the body and the left and right sides, where *i* indicates the tail beat cycle number. The phase lag is defined as

$$\begin{array}{c} \phi_{i}^{L}=\frac{t_{act,i}^{L}-t_{curve,i}^{L}}{t_{act,i+1}^{L}-t_{act,i}^{L}}, \end{array}$$ (19)

for each side of the body, so that a positive value of the phase lag indicates that the muscle is active while shortening and a negative value indicates that muscle is active while lengthening. In this convention, phase runs from 0 to 1.

We then computed the activation wave speed *v*_*a*_ and the curvature wave speed *V*. Activation waves travel in anti-phase down the left and right chains of oscillators, at the same speed. For simplicity, we write the equations below for phase *θ* (chosen on one side) and curvature *κ* defined continuously on position *s* and time *t*. In the analysis, we use central differences to approximate the temporal and spatial derivatives. The activation wave speed is defined based on the oscillator phase *θ* (Eq (1)),

$$\begin{array}{c} v_{a}\left(s,t\right)=\frac{\partial \theta (s,t)/\partial t}{\partial \theta (s,t)/\partial s}. \end{array}$$ (20)

To compute curvature wave speed accurately, it is best to estimate a curvature phase using the Hilbert transform *H*{⋅} [46], an operator that estimates the complex representation of a real-valued oscillatory signal like curvature *κ*(*s*, *t*), such that |*H*{*κ*(*s*, *t*)}| is the curvature amplitude and *ϕ* = ∠*H*{*κ*(*s*, *t*)} is the phase of the curvature, both at a position *s*. Then the curvature wave speed is

$$\begin{array}{c} V=\frac{\partial \phi (s,t)/\partial t}{\partial \phi (s,t)/\partial s} \end{array}$$ (21)

and the wavespeed ratio is 〈*V*〉/〈*v*_*a*_〉, where 〈⋅〉 indicates a mean over time and space. Specifically, we take the mean over the region *s* ∈ (0.5…0.8) and over the last 7 tail beats.

When the ratio is less than one, it indicates that the curvature wave is travelling more slowly than the activation wave (as in Fig 6). The difference in wave speed means that the curvature wave increasingly lags behind the activation wave as the two move down the body, with the phase lag then becoming larger in magnitude and more negative toward the tail, the same pattern seen in fishes [37] and in our previous model [20].

## Results and discussion

### Control case without feedback

As a baseline control case, we examined our model without feedback. The muscles in the model are activated by a CPG, which is approximated as a set of phase oscillators that receive input from each other and from the body’s curvature through a feedback function. When the feedback function is zero (*η*(*κ*_*i*_) ≡ 0), there is no feedback and the phases evolve with no dependence on body curvature. This corresponds to the gain *η*_*m*_ = 0 or *η*_*d*_ = 0 in each of the two feedback models that we propose. For this control case, because the phases of segments *i* and *j* on each lateral side of the lamprey body are initialized to differ by the prescribed phase shift *ψ*_*ij*_ and the phases of segments on opposite sides are initialized to differ by *π*, all phases move at the constant velocity *ω*. The initial conditions chosen for the phase oscillators did not affect the steady state swimming results, as long as the chains were not initialized with exactly the same phases. To maintain consistency in the simulations, the same initial conditions were used for each simulation.

To produce a muscle activation signal, the phases are used to construct an on-off activation following Eq (3). The cutoff value *τ* was tuned so that duration of the activation signal in the control case matches that of freely swimming lampreys [4] and of our previous models [19, 20]. This activation signal has a temporal period of one second and a duty cycle of 0.36, meaning that each segment is activated for 36% of the period. Because we tuned the neural activation signal to be the same as in our previous models, and because the body and fluid mechanics are the same, the emergent kinematics and energetics of this control case match the computational results in our previous work [19, 20].

**Fig 7:**
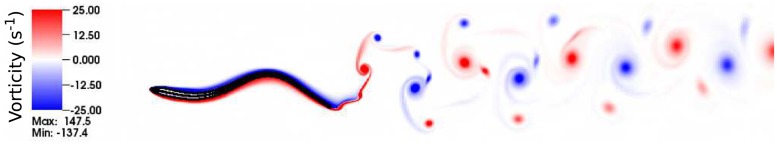
Simulation with no feedback. The body kinematics and wake structure for the control case is shown at *t* = 8.0 s. The outline and midline of the computational lamprey are shown in black. Vortices shed from the tail are shown in red (counterclockwise rotation) and blue (clockwise rotation). This model lamprey swims at a speed of 0.52 L · s^−1^ with a tail beat amplitude of 0.12 L and a body wavelength of 0.75 L. Natural lampreys swim at about 0.1 L/s (body lengths per second) during migration [48] with maximum sustained speeds of about 2.5 L/s [49].

Fig 7 shows a snapshot of the lamprey body and vorticity wake for this control case. The inputs to this model system are the passive bending rigidity of the lamprey, the fluid viscosity and density, the maximum tetanic force that each muscle could exert, the baseline frequency and coupling parameters of the CPG, and the activation cutoff value. The outputs are the flow patterns (shown as red and blue vorticity in Fig 7), the evolution of the CPG phase values, and the movement of the body (black lines in Fig 7). From the body movement, as in previous studies, we compute the swimming speed and the amplitude, wavelength, and speed of the mechanical wave [19, 20, 39], the ratio of the wave speeds of the mechanical and activation waves, [37, 47], and the muscular work done by swimmer. Based upon swimming velocity, body length and the density and viscosity of water, the Reynolds number of this control swimmer is about *Re* = 8000.

In previous studies using this model, we examined how the swimming performance depended upon body stiffness [20] and muscle nonlinearities [19]. While one could have used shear as a body-sensing measure, in the closed-loop model presented here, we explore how different assumptions about feedback from body curvature on the evolving signal from the CPG affect this swimming performance.

### Case 1: Effects due to directional feedback (D)

In the lamprey, proprioceptive feedback is mediated by mechanoreceptive cells in the spinal cord called edge cells [5]. When the spinal cord bends, one side is stretched, which activates edge cells on that side [5, 13]. The greater the curvature, the stronger the activation [13]. The activated edge cells then inhibit activity on the opposite side and excite activity on the same side [7]. To approximate these effects in our model, we assume that the feedback signal is proportional to the magnitude and direction of curvature. For these simulations, labeled D, *η*(*κ*_*i*_) = (−1)^*k*^ *η*_*d*_ *κ*_*i*_ where *k* is the side of the body (1 is left, 2 is right). Thus, the natural case is represented by *η*_*d*_ > 0. If *κ*_*i*_ is positive, representing bending to the right, and *η*_*d*_ is positive, oscillators on the right side of the body receive a positive signal, representing excitation, while those on the left side receive a negative signal, representing inhibition.

**Fig 8:**
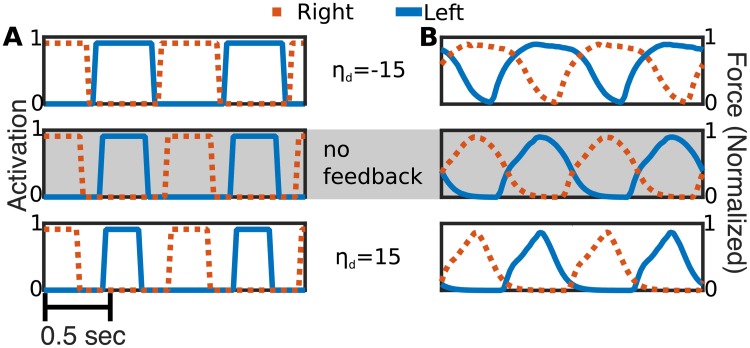
Activation and force with directional (D) feedback. Two seconds of simulated time 44% of the way down the body from the tail of the lamprey body at different indicated gains. The blue solid line and red dotted line are the left and right sides of the body, respectively. **A:** Activation state for the segment and each feedback case. “0” indicates the segment is inactive, “1” indicates the segments is active. **B:** Forces developed on the segments, scaled by the maximum tetanic force.

This class of feedback affects the duty cycle during steady swimming, without affecting the frequency. Each swimmer was initialized in a straight configuration in a still fluid near the right of the domain. Fig 8(A) shows the computed activation signal over two seconds of simulated time on each side of a representative segment of the lamprey body for gains *η*_*d*_ = −15 cm · rad · s^−1^ (top), *η*_*d*_ = 0 cm · rad · s^−1^ (middle), and *η*_*d*_ = 15 cm · rad · s^−1^ (bottom). For instance, for the positive gain value, the amount of time that a muscle segment is activated is less than that for the control case. Fig 8(B) shows the corresponding force development on the opposing segments for each of the simulations. The high duty cycle in the negative gain case lead to considerable co-contraction, as seen by the overlapping blue and red curves on the top panel in Fig 8B. The amount of co-contraction decreases as gain increases.

**Fig 9:**
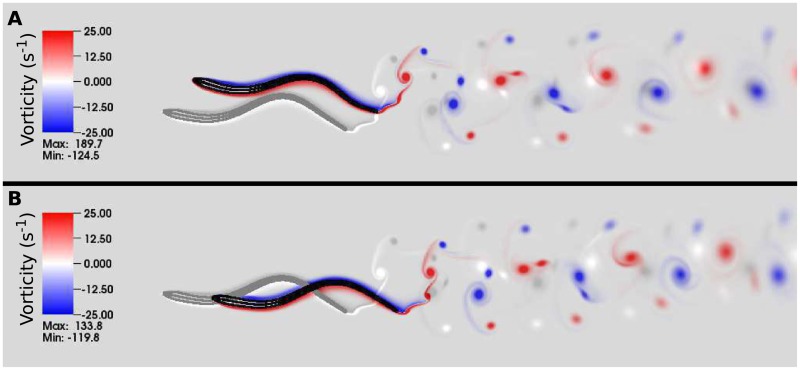
Effects of directional feedback. Body configuration and trailing wake structures for lamprey simulations after 8 s of simulated time. The control case (without feedback) is shown in gray. Panels **A** and **B** show the effects of feedback negative and positive gains (*η*_*d*_ = −15 cm · s^−1^ and 15 cm · s^−1^, respectively), and the corresponding dynamics are shown in S1 and S2 Movies.

Fig 9 shows how these differences in force development on individual segments affect the overall swimming kinematics and dynamics. Whether the gain is positive or negative, each swimmer with directional curvature feedback (in black) swims more slowly than the control (gray). The dynamics of the negative gain swimmer compared to the control is shown in S1 Movie, and the dynamics of the positive gain swimmer compared to the control is shown in S2 Movie. In the case of negative gain, the long duration of co-contraction of opposing segments reduces the achieved tail-beat amplitude of the swimmer. In the case of positive gain, the short duration of activity leads to less total force, which also reduces the achieved tail beat amplitude. These effects are quantified in detail below.

### Case 2: Effects due to magnitude feedback M

**Fig 10:**
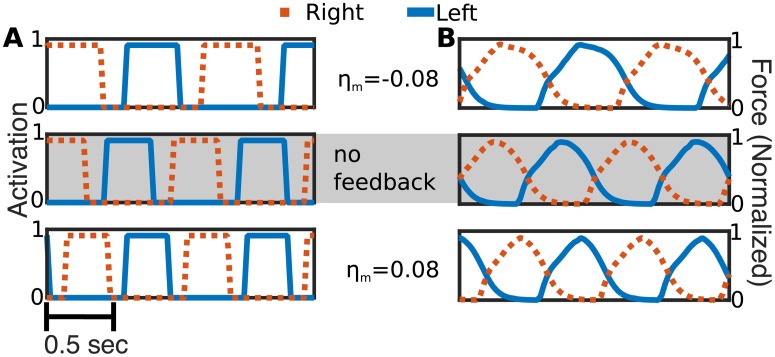
Activation and force with magnitude only magnitude feedback. Two seconds of simulated time at 44% down the lamprey body at different indicated gains. The blue solid line is the left side of the body, the red dotted line is the right side of the body. **A:** Activation state for the segment and each feedback case. “0” indicates the segment is inactive, “1” indicates the segment is active. **B:** Forces developed on the segments, scaled by the maximum tetanic force.

In the lamprey, activating edge cells produce a generalized excitatory effect on the CPG [41, 42] that does not depend on the direction of the curvature. To approximate this effect, we examined feedback that depended only on the magnitude of curvature. In this case, labeled M, the feedback advances the phase of the oscillators equally on both sides. For a fixed muscle segment, the feedback term *η*(*κ*_*i*_) = *η*_*m*_|*κ*_*i*_| increases the frequency of the activation signal if *η*_*m*_ is positive and decreases it if *η*_*m*_ is negative. Fig 10(A) shows the computed activation signal over two seconds of simulated time on each side of a representative segment of the lamprey body for gains *η*_*m*_ = −0.08 cm · rad · s^−1^ (top), *η*_*m*_ = 0 cm · rad · s^−1^ (middle), *η*_*m*_ = 0.08 cm · rad · s^−1^ (bottom). For each gain, we see that the activation signals on the left and right sides are in opposite phase (when one side is on, the other is off) and the duty cycle is approximately constant. Unlike the D case, we see that frequency of activation increases with gain. Fig 10(B) shows the magnitude of the contractile force exerted by the opposing segments that results from the activation signals shown in Fig 10(A) for each gain value. We see the maximal contractile force achieved is basically unchanged in each case, but the frequency increases with gain so that the force is maintained for a shorter period of time. This reduces the total force developed in each cycle. Hence, in the positive gain case the total activation time is lower, so muscles achieve peak force for less time (Fig 10B, bottom panel).

**Fig 11:**
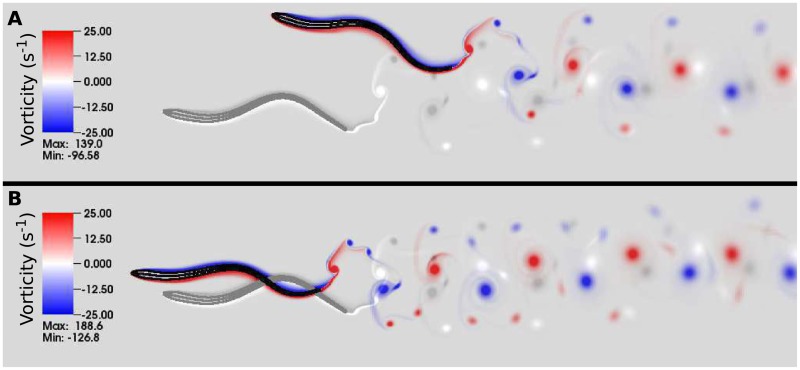
Effects of feedback due to curvature magnitude. Body configuration and trailing wake structures for lamprey simulations after 8 s of simulated time. The control case (without feedback) is shown in grey. Panels **A** and **B** show the effects of feedback with negative and positive gains (*η*_*m*_ = −0.08 cm · rad · s^−1^ and 0.08 cm · rad · s^−1^, respectively) and the corresponding dynamics are shown in S3 and S4 Movies.

With this class of feedback, swimmers with positive gain swim faster than the control case, while those with negative gain swim slower. Fig 11 shows the body shape and flow patterns for two swimmers at time *t* = 8 s, one with gain *η*_*m*_ = −0.08 cm · rad · s^−1^ (Fig 11A) and the other with gain *η*_*m*_ = 0.08 cm · rad · s^−1^. The dynamics of the negative gain swimmer compared to the control is shown in S3 Movie, and the dynamics of the positive gain swimmer compared to the control is shown in S4 Movie. While each swimmer progressed towards the left as the waves of neural activation moved from head to tail, the swimmer with negative gain progresses more slowly than the control because of the decreased beat frequency. Its drift upwards is due to transient fluid effects from the strong start-up vortex that is shed during the first tail beat. Because of the lamprey’s reduced beat frequency and the subsequent reduced swimming velocity, this vortex remained near the body, pushing it upwards. The emergent waveforms of the three swimmers differ only slightly in amplitude and wavelength.

### Effects of feedback on activation signal

In the examples shown above, we see that magnitude feedback (M) affects the frequency of the activation signal, while directional feedback (D) alters the duty cycle. Here we examine how these changes in activation signal depend upon the gain constants *η*_*m*_ or *η*_*d*_. As the phase model is heuristic, there are no direct experimental measurements that can be used to set the values for these constants. We performed computational experiments to determine the range of values of the gains around the control case of *η*_*m*_ = 0 or *η*_*d*_ = 0 that resulted in sustained, periodic swimming. For instance, for large absolute values of gain, the phase velocities could either get too large, or, in the case of directional feedback, the phase velocities could become negative, so that realistic activation signals propagating along the left and right muscle segments could not be achieved. For the case of magnitude feedback, we found the range to be |*η*_*m*_| < 0.09 cm · rad · s^−1^, and for directional feedback the range was found to be |*η*_*d*_| < 20 cm · rad · s^−1^. To compare the effects of the different classes of feedback at a range of gains, we scaled the gain relative to the maximum range for that class. In other words, we scaled *η*_*m*_ by 0.09 cm · rad · s^−1^ and *η*_*d*_ by 20 cm · rad · s^−1^.

**Fig 12:**
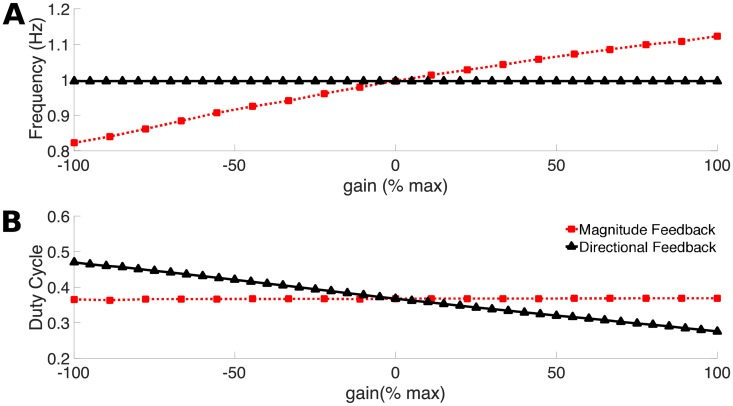
Effects of feedback on tailbeat frequency and duty cycle. **A**: The tailbeat frequency plotted against the percent gain.**B**: The average duty cycle (ratio of active cycle time to total cycle period) averaged along the body for 6 steady swimming cycles plotted against the percent gain.

The frequency of the CPG activation signal on each muscle segment directly affects the tailbeat frequency of the computational swimmer. Fig 12A shows the tailbeat frequency as a function of the non-dimensional gain parameter. We see that in the magnitude feedback case, tail beat frequency increases linearly with gain. However, in the directional feedback case, the frequency is unchanged from 1 Hz. In contrast, Fig 12B shows the average duty cycle along the lamprey body as a function of non-dimensional gain. While the duty cycle is nearly constant at 0.36 in the magnitude feedback case, it decreases linearly with gain in directional feedback. We examine the implications of these effects on body kinematics and swimming performance below.

In living fishes, both frequency and duty cycle vary. Nearly all fishes increase tail beat frequency as they increase swimming speed while maintaining a fairly constant tail beat amplitude [31, 50]. Duty cycle varies along the body and across fish species [4, 37], and a recent study shows that, in bluegill sunfish, it decreases with increasing swimming speed [51].

Our results suggest that feedback structure may modulate frequency and duty cycle, but fish can alter activation frequency and duty cycle in a variety of other ways. Frequency may be modulated by changing the descending drive from the brain [52, 53]. Less is known about how duty cycle may be modulated. Currently only the phase of the oscillators and not the amplitude of the activation are affected by feedback. This type of model will capture changes in frequency of activation and deactivation, but not the relative strength of those activations. In the future we will incorporate models in which the amplitude of the CPG output may be affected by feedback input, which would require shifting from a phase model to a model that incorporates neural properties (e.g. [33, 54]).

The response to sensory feedback is much more sensitive to the magnitude of curvature, rather than the magnitude and direction. This is due to an antisymmetry in the directional signal across each segment of the body. Positive curvature feedback from one side is partially cancelled by negative curvature feedback from the other side, or *vice versa*. When feedback is only due to the magnitude of the curvature, the feedback signals from both sides tend to add, making the overall system much more sensitive to this type of feedback.

### Effects of feedback on body amplitude and wavelength

**Fig 13:**
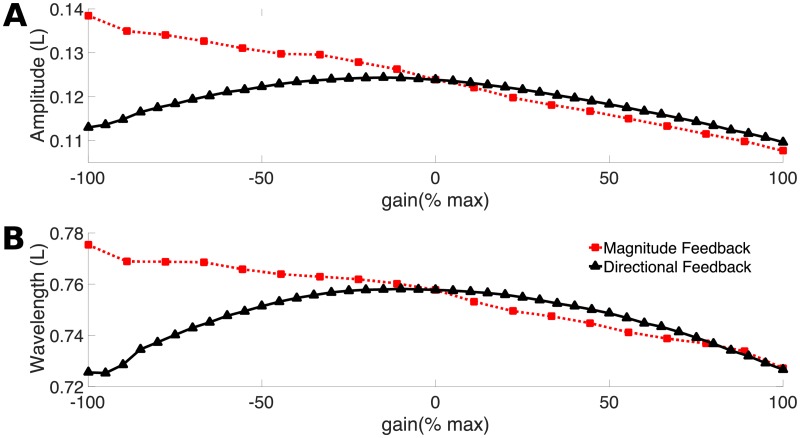
Effects of feedback on lamprey waveform: Amplitude and wavelength. **A:** Tailbeat amplitude and **B:** curvature wavelength as a function of the non-dimensional gain. Both amplitude and wavelength are reported in units of the body length *L*. Each simulation was run for 10 s of simulated time, and the values reported are averaged over the beat periods when steady swimming was achieved.

Both forms of feedback change the duration of the activation signal, either symmetrically for both left and right sides (magnitude feedback) or asymmetrically (directional feedback). Because the muscle model requires time to develop force, the shorter the activation duration, the lower the average force. For example, compare the top and bottom panels in Fig 10B. These contractile forces are coupled to the body’s passive elastic forces and to the viscous incompressible fluid surrounding the lamprey. Together, this coupling leads to an emergent swimming waveform. Fig 13 shows the tail beat amplitude and body wavelength for the computational lamprey as a function of non-dimensional gain.

Fig 13A shows that the two types of feedback have qualitatively different effects on amplitude. In the magnitude feedback case the amplitude steadily decreases as the gain increases. The decrease in tail amplitude is due to the increase in activation frequency, which reduces the average force developed at a segment in each cycle. In the directional feedback case, tail amplitude is no longer monotonic with gain, but instead has a maximum at a slightly negative gain. With the most negative gains, the considerable amount of co-activation offsets the increased force development so the net force available for bending is reduced, resulting in a lower amplitude. At high positive gains, the duration of muscle activity is very low, which means that the muscle cannot produce much force, which also results in a low amplitude (see Fig 8). In between, at gain *η*_*d*_ = −3 cm · rad · s^−1^ (−15%), the amount of co-activation is optimal, resulting in the largest amplitude.

For both classes of feedback, wavelength follows a very similar pattern as amplitude (Fig 13B). It decreases with increasing gain for magnitude feedback, and has an optimum for directional feedback. Because the lamprey body is nearly inextensible, it may seem inconsistent for amplitude and wavelength to follow the same pattern. For a traveling wave with a constant amplitude along the body, wavelength should be inversely proportional to amplitude. However, here we are reporting only tail amplitude. In fact, the wave envelope as a whole depends nonlinearly on the feedback, and inextensibility is maintained. For example, at negative gains for magnitude feedback, as tail amplitude increases the head amplitude decreases, which allows the wavelength also to increase even as the body length stays the same.

### Effects of feedback on swimming speed

**Fig 14:**
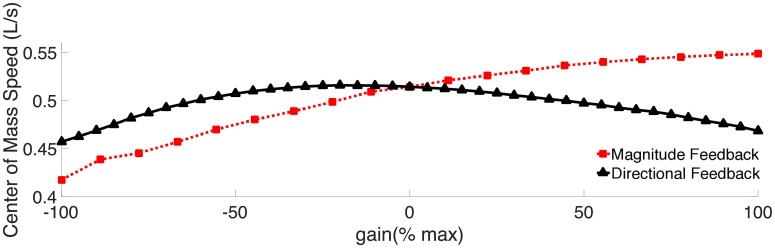
Changes in swimming speed with gain. Center of mass speed in L · s^−1^ averaged over *t* = 6 s to 10 s of simulated time.

The body deformation couples with the surrounding fluid to give rise to forward movement. Natural lampreys swim at about 0.1 L/s (body lengths per second) during migration [48]. Maximum sustained speeds are about 2.5 L/s [49] and they are capable of bursts up to about 5 L/s [49]. Fig 14 shows the swimming speed of the computational lamprey as a function of non-dimensional gain for both the directional feedback cases. The swimming speed for magnitude feedback increases as gain increases, but begins to level off at the most positive gain values. This effect is due to the combined effects of tail beat frequency and amplitude. As gain increases, frequency increases but amplitude decreases, both approximately linearly. In swimming eels, swimming speed is best correlated with the lateral tail velocity, or *fA*, where *f* is frequency and *A* is amplitude [39]. Thus, if *U* ∝ *fA* for our swimmer, the relationship should be nonlinear, which is what is seen in Fig 14.

Fig 14 also shows that for the directional feedback case, the effects of gain on swimming speed are similar to those on amplitude, since frequency is unchanged. However, even though the shapes of the curves are similar, the swimming speed is not a simple function of the tail amplitude, because the maximum swimming speed does not occur at the same gain as the maximum amplitude. The maximum swimming speed, 0.52 L · s^−1^, occurs at a gain of *η*_*d*_ = −4 cm · rad · s^−1^ (−20%), corresponding to a duty cycle of 0.389, while maximum amplitude occurs at a gain of −3 cm · rad · s^−1^ (−15%), corresponding to a duty cycle of 0.3831.

### Effects of feedback on Strouhal number and phase lag

**Fig 15:**
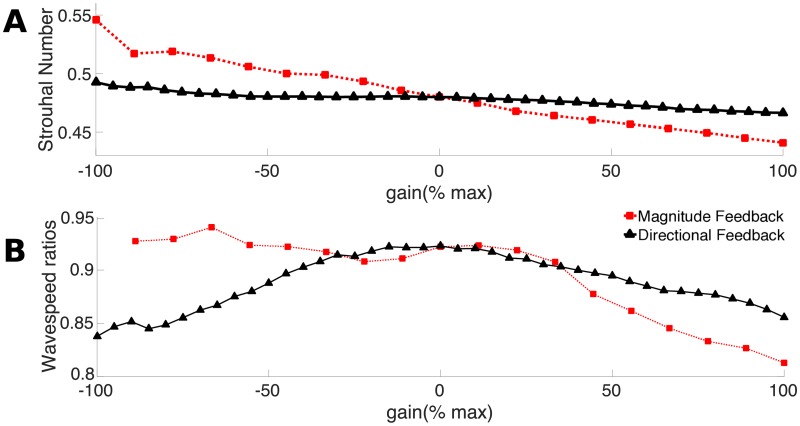
Changes in Strouhal number and phase lag with gain. **A:** Mean Strouhal number calculated for vortex shedding over *t* = 6 s to 10 s of simulated time. **B:** The slopes of the phase lags calculated for each gain according to Fig 6.

A measure of the effectiveness of a swimming stroke is the Strouhal number *St* which is the ratio of the speed of the tail to the forward swimming motion

$$\begin{array}{c} St=\frac{2fA}{U} \end{array}$$ (22)

where *f* is the tailbeat frequency, *A* is the tailbeat amplitude and *U* is the swimming speed. Lower *St* indicates a more effective swimming motion, because the tail’s side-to-side velocity is lower relative to the forward velocity. Fig 15 shows that for both directional feedback cases, *St* decreases as non-dimensional gain increases, although the total change in *St* is larger for the magnitude cases than the directional cases. The swimmer is thus becoming a more effective swimmer as gain becomes larger and positive. Even so, compared to swimming fishes, the Strouhal number is still relatively high. Fishes tend to swim with *St* between 0.2 and 0.4 [55, 56], which corresponds to an efficiency optimum [56]. We note that since our model is 2D, agreement between computed Strouhal numbers and those of natural animals is qualitative, however the 2D model is an essential step to understanding swimming in a 3D fluid.

Another metric associated with effective swimming is the neuromechanical phase lag [37, 38, 57]. At anterior points on the body, once the swimmer reaches steady state, the bending precedes muscle activity, so that muscle activity is always while the muscle is shortening. When a muscle is active while shortening, it produces positive mechanical power. Closer to the tail, the muscle becomes active earlier in the cycle, sometimes to the extent that the muscle is active while lengthening (see Fig 6 for an illustration of this effect) [47]. When the posterior muscles are active while lengthening, they absorb energy, producing negative mechanical work. At the same time, these muscles also stiffen the posterior body, which helps to transmit body forces to the fluid more effectively [20, 38]. In Fig 6, we quantify this effect by computing the ratio of the mechanical wave speed and the activation wave speed. When this ratio is less than one, it mean that the posterior muscle is active earlier in the cycle, similar to what is seen in fishes.

Fig 15B shows that for the magnitude feedback case, the wavespeed ratio decreases with increasing gain. This change is approximately in proportion to the increase in frequency (Fig 12A) and the associated decrease in body wavelength (Fig 13B). In this feedback case, the activation wave has the same wavelength, regardless of gain, which explains the decrease in the wavespeed ratio.

In the directional feedback cases, the wavespeed ratio is not monotonically associated with gain (Fig 15B). Instead, it comes closest to one at *η*_*d*_ = −20%, similar to the effect of gain on curvature wavelength (Fig 13B). At high positive or negative gain, the ratio decreases, indicating a larger phase lag near the tail for these cases. In this case, however, the effect is not solely due to the change in curvature wavelength. At high negative gain, the activation wave is longer (1.24 L), while at high positive gain, the wave is shorter (0.72 L). The change in activation wavelength accounts for the smaller decrease in the ratio at positive gains with directional feedback (Fig 15B).

These results are partially consistent with the hypothesis we proposed for the earlier model, that higher phase lags develop when the internal mechanical forces are low relative to the fluid forces [20]. For the magnitude feedback case, increasing frequency results in lower average muscle force (Fig 10), even as swimming speed increases, which suggests that the internal forces are declining relative to the external forces. For the directional feedback case, the same effect is seen for positive gain: duty cycle decreases, so the average muscle force decreases (Fig 8B), leading to a decrease in internal muscle forces relative to external fluid forces.

The small wavespeed ratio for negative gains in the directional case seem to follow a different pattern. The internal muscle forces increase on average for negative *η*_*d*_ (see Fig 8) and there is more co-activation, which should lead to an effectively stiffer body overall [58]. The activation wavelength, though, gets longer at more negative gain, even as the curvature wavelength gets shorter. This leads to a decreasing wavespeed ratio.

Williams *et al*. [47] measured the wavelengths of the mechanical and activation waves in lampreys. Based on her published results, lampreys swim with a wavespeed ratio of approximately 0.7 [47], a value that is substantially lower than we find in any of our simulations (Fig 15B). Experimental results suggest that lampreys may respond differently to mechanical inputs in the posterior part of the body [9, 10], a pattern that may increase the phase lag near the tail [9]. In our simulations, sensory inputs are processed equivalently, regardless of their location. To accurately reproduce the phase lag of living lampreys, we may need to alter the feedback pattern along the body.

### Effects of feedback on cost of transport

We approximated the cost of transport (metabolic energy per unit length per unit distance). Muscles produce positive mechanical work when they are active and shortening, and require a metabolic energy input that is proportional to the mechanical work. Muscles produce negative mechanical work when they are active and shortening. This process requires much less metabolic energy, but is still proportional to the amount of negative work (Ruina et al. [59]). Thus, following Hamlet et al. [19], we compute the cost of transport by summing the positive work and a fractional amount of the absolute value of the negative work done by the lamprey’s muscles over each segment at each time step over a full cycle, and then divided the result by the cycle period and mass of the lamprey (as in [19]). For comparison, we also normalized the results by the cost of transport for the control case.

**Fig 16:**
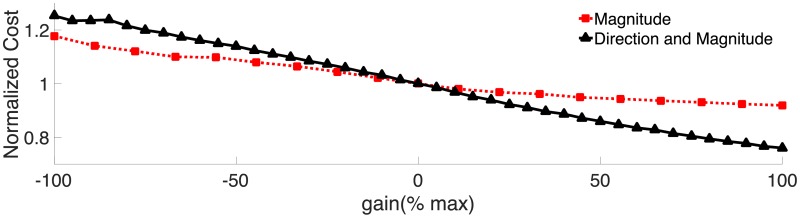
Effects of feedback on cost of transport. The cost of transport calculated for each gain, normalized by the cost for the control case without feedback.

All of the kinematic and muscular factors described above combine to propel the swimmer forward with a particular energy cost to go a certain distance. For both feedback cases, the cost of transport decreases with increasing gain. Fig 16 shows the normalized cost of transport as a function of non-dimensional gain. The effect is most pronounced for the directional feedback case. At the highest positive gain, the swimmer uses 24% less energy to go the same distance as the control case. For the magnitude feedback case, the effect is less linear. At the highest positive gain, the swimmer uses 8% less energy, but at the most negative gain, the swimmer uses 18% more energy.

For both feedback cases, the average muscular force decreases at higher positive gain (Figs 8B and 10B), resulting in lower average muscular work. The Strouhal number also decreases, indicating that these swimmers are hydrodynamically more effective. Thus, the overall cost of transport decreases as gain increases.

At negative gain, the cost increases, but the cause of the increase is different for the two feedback cases. For magnitude feedback, the increase in cost is due to the same effect that causes the decrease for positive gain. As gain decreases and becomes negative, the muscular work increases and Strouhal number increases, indicating a less effective swimmer with a higher overall energetic cost. For directional feedback, the increase in cost is due to the increase in co-activation of the left and right side muscles. As gain becomes more negative, more and more of a muscle’s force is used to oppose its antagonist, rather than deforming the body and producing forward propulsion.

Wavespeed ratios less than one, which correspond to negative phase lags near the tail, have been thought to indicate more effective swimming [38], possibly with lower cost of transport. Our simulations suggest that this is not always true. In the directional feedback case, the wavespeed ratio becomes smaller as gain becomes negative, but the overall cost goes up, due to the increase in co-activation.

Cost of transport does not explicitly include speed. In both feedback cases, at high positive gain, the swimmer uses less energy to go a unit distance. However, the swimmer with magnitude feedback goes 17% faster than the swimmer with directional feedback, so it takes less time to go that distance. Animals that need to minimize energy cost may need to factor in both the overall cost of transport and also the speed.

### Comparison to other simulations

Many studies of feedback examine its role in responding to perturbations. Our simulation, in contrast, examines the role of feedback in maintenance of a steady effective locomotor pattern. Relatively few other studies have taken this approach. For example, Ekeberg and Grillner [28] developed a neuromechanical model of lamprey swimming that incorporated feedback from edge cells, proportional to curvature, similar to our directional feedback case. They found a “slight increase” in spatial wavelength, within the range of uncertainty in the parameters, when edge cell feedback was included [28], but they did not investigate whether the effects might be different if the gain was higher. It seems likely that their model was in the range of low gain feedback in which wavelength does not change substantially with gain (i.e., −20 < *η*_*d*_ < 40 in Fig 13B).

Gazzola et al. [31] simulated a minimal model of a flexible swimmer that included proprioceptive feedback. They were interested in whether feedback alone, without a CPG, could allow the model to swim steadily. They included passive elastic forces based on a beam model, active muscular forces as an additional internal torque, and hydrodynamic forces based on elongated body theory. They modeled proprioceptive feedback by adding a term to the internal torque. This feedback term was directly proportional to local curvature, except with a time delay. With a CPG, but without feedback, they found that the swimmer had a resonant behavior: it had peaks in swimming speed at several activation frequencies. With feedback, but without the CPG, they found that the swimmer tended to converge to the nearest resonant peak, as long as they gave it some starting deformation. They found that increasing the feedback gain (*χ* in their Eq [9]), tended to increase the amplitude and the swimming speed. It is difficult to compare that finding to our results, because of the lack of a CPG model in their feedback case, but their feedback term is most similar to our directional feedback model. Unlike their result, we found that amplitude and swimming speed decreased as we increased feedback gain. This difference may be related to changes in level of co-activation in our model, a property that cannot be simulated directly using an added torque as in Gazzola et al. [31].

Very recently, Thandiackal and Ijspeert [60] simulated a similar swimmer, using a CPG modeled with phase oscillators and a feedback term that depended on the local fluid dynamic pressure. Their fluid dynamic model is based on elongated body theory [61], which has been validated, but is much simpler than our Navier-Stokes model. They modeled their feedback approach on the lateral line sensory system in fishes, while ours is modeled on the edge cells in lampreys. In their model, the feedback had a very strong effect on both the mechanical and activation wavelength. In our model, without feedback, the system will have an nominal activation wavelength of 2*π*/*ψ* (see our Eq (1) and their Eqn. 2.3). In their model, as they varied *ψ* over a wide range, feedback tended to push the swimmer to have about one full wave on its body. If the nominal wavelength was small, feedback increased the wavelength, and if the nominal wavelength was large, feedback decreased the wavelength [60]. We did not examine the effects of changing *ψ*, but we also found that feedback could have strong effects on wavelength.

### Biological implications

Our feedback model approximates two different known effects in the lamprey’s sensorimotor control system: first, the phasic effect of edge cells [7] that tends to enhance the ongoing locomotor rhythm [2], and second, the generalized excitatory effect of sensory feedback [3, 41]. Even in the absence of perturbations, we find that both effects are important during steady locomotion.

In our model, both forms of feedback primarily affect the duration and frequency of muscle activation along the body. Increasing the frequency of activation (through magnitude feedback) increases the swimming speed, as seen in fishes [39, 50]. In fishes, however, amplitude usually remains approximately constant as frequency and speed increase [50]. In our model, the amplitude decreases as frequency increases (Fig 13), which means that speed does not increase linearly (Fig 14). This suggests that fish probably have to increase the overall activation strength as frequency goes up, in order to increase swimming speed.

Increasing the duration of muscle activity (through directional feedback) changes the amount of co-activation of muscles on opposite sides of the body. This has a more complex effect on swimming than changing frequency. In particular, the fastest swimmers have a fairly substantial amount of co-activation (Fig 14, black curve). Co-activation can change the effective stiffness of a joint [62], and our previous results showed that there is an optimal passive stiffness for maximum swimming speed [20]. It may be that the co-activation we see here alters the effective stiffness to maximize swimming speed. However, co-activation also comes at a cost. Swimming at the optimal speed requires more energy than swimming at lower speeds with less co-activation (Fig 16, positive gains).

### Conclusions

Sensory feedback may have an important ongoing effect on steady locomotion, even in the absence of perturbations. With both forms of curvature feedback, we see that the energetic cost of transport decreases with gain. The decrease in cost may also be aided by the decrease in force with gain (Fig 10), which in turn decreases the muscular work at higher gains. However, speed increases with increasing gain (Fig 14) and Strouhal number decreases, which indicate that swimming is becoming more effective at higher gain, leading to an overall decrease in energetic cost.

While it is well-established that the lamprey’s edge cells are stretch receptors, exactly how this information on stretch feeds back to the CPG is not known. As a starting point to explore the closed-loop locomotor system, here we chose a simple phase-oscillator model of the CPG as well as simple models of feedback where curvature has an additive effect on the evolution of the phase oscillators. In the phase oscillator model, the feedback can only affect the phase of the CPG signal and not its amplitude. More detailed CPG models that capture the neural system (e.g. [45, 63]) will be necessary to analyze these effects. Within the context of the simple model presented here, future investigations will study the effect of feedback with a time delay, as well as the effect of Reynolds number on the system where, perhaps, an animal may need to change it’s control strategy as it grows. In addition, here we only examined the closed-loop system during steady locomotion in the absence of perturbations. Future studies will investigate how the closed-loop swimmer reacts to perturbations in the fluid environment as well as how curvature feedback affects it’s ability to actively turn.

## Supporting information

S1 Fig Figures showing the waveforms and amplitude envelope for specific cases of feedback discussed in the main text. The bottom plot shows the control “no feedback case”. Each figure shows the waveforms for t = 5s to t = 6s plotted at regularly spaced intervals.
(EPS)

S2 Fig Close up of the head (A) and the tail (B) of the lamprey. Each set of point is numbered head to tail. In this figure, the top set of points is referred to as the “right side” of the body. The body is constructed with an ellipse-shaped head whose cross-section increases for points 1-20. In this “head” region, there no muscle activation is imposed. The cross-sectional width of the lamprey is a maximum of 10% the body length at point 20 (A), then linearly decreases to a width of 0.1% of the body length at point 321 (B).
(EPS)

S3 Fig Values for coupling strengths *α*_*i*,*j*_, between oscillators as calculated using parameters from the Table in S1 Table in the supplemental information and Eq 2 in the main text. The strengths of the ascending ((*i* − *j*) < 0) and descending ((*i* − *j*) > 0) connections as a function of the number of segments between the points. The blue line shows the descending strengths, while the red line shows the ascending strengths.
(EPS)

S1 Movie Comparison of the dynamics of a swimmer with feedback due to directional feedback (D) with control swimmer. Here gain is negative (*η*_*d*_ = −15 cm · s^−1^). The body configuration and trailing wake structures for the swimmer with feedback are shown after 8 s of simulated time. The control case (without feedback) is shown in gray.
(MPG)

S2 Movie Comparison of the dynamics of a swimmer with feedback due to directional feedback (D) with control swimmer. Here gain is positive (*η*_*d*_ = 15 cm · s^−1^). The body configuration and trailing wake structures for the swimmer with feedback are shown after 8 s of simulated time. The control case (without feedback) is shown in gray.
(MPG)

S3 Movie Comparison of the dynamics of a swimmer with feedback due to magnitude feedback (M) with control swimmer. Here gain is negative (*η*_*m*_ = −0.08 cm · s^−1^). The body configuration and trailing wake structures for the swimmer with feedback are shown after 8 s of simulated time. The control case (without feedback) is shown in gray.
(MPG)

S4 Movie Comparison of the dynamics of a swimmer with feedback due to magnitude feedback (M) with control swimmer. Here gain is positive (*η*_*m*_ = 0.08 cm · s^−1^). The body configuration and trailing wake structures for the swimmer with feedback are shown after 8 s of simulated time. The control case (without feedback) is shown in gray.
(MPG)

S1 Table Parameters used in the muscle and body models. Calcium and force parameters were fitted to data provided courtesy of T. L. Williams; velocity and length dependence, and muscle stiffness parameters are from Williams [22] and McMillen et al. [23].
(PDF)

S1 Text Equations describing the springs used to model the passive regions of the computational lamprey body. (PDF)
