# Supplementary material for: The role of curvature feedback in the energetics and dynamics of lamprey swimming: A closed-loop model
Source: PLoS Comput Biol. 2018 Aug 17;14(8):e1006324. doi: 10.1371/journal.pcbi.1006324 (PMC6114910; doi:10.1371/journal.pcbi.1006324)
Supplement: S1 Table — Calcium and force parameters were fitted to data provided courtesy of T. L. Williams; velocity and length dependence, and muscle stiffness parameters are from Williams [22] and McMillen et al. [23]. (PDF) [file pcbi.1006324.s008.pdf]

Table S1

Parameter	Value	Dimensions
**velocity dependence**		
α_m	0.80	s
α_p	2.90	s
α_{max}	1.8	
α_{min}	0	
**length dependence**		
λ_2	-20	
λ_{max}	1	
λ_{min}	0	
**muscle stiffness**		
μ_0	1	
μ_1	23	
**force parameters**		
P_0	60.86	$\mathrm{mN} \cdot \mathrm{mm}^{-2}$
m_s	0.0542	s^2
d_s	0.2802	s
l_{c0}	0.91	
**calcium parameters**		
k_1	6.7281	s^{-1}
k_2	23.2794	s^{-1}
k_{30}	51.3537	s^{-1}
k_{40}	19.3801	s^{-1}
k_{m1}	17.5804	
k_{m2}	6.0156	s^{-1}
C	2	
S	6	
**body parameters**		
L	varies	cm
L_0	varies	cm
s_{skin}	10.24	$\mathrm{N} \cdot \mathrm{cm}^{-2}$
s_{int}	25.6	$\mathrm{N} \cdot \mathrm{cm}^{-2}$
**oscillator coupling parameters**		
A_a	1.0	$\mathrm{rad} \cdot \mathrm{s}^{-1}$
A_d	10.0	$\mathrm{rad} \cdot \mathrm{s}^{-1}$
λ_a	40	
λ_d	5	
