# Supplementary material for: The role of curvature feedback in the energetics and dynamics of lamprey swimming: A closed-loop model
Source: PLoS Comput Biol. 2018 Aug 17;14(8):e1006324. doi: 10.1371/journal.pcbi.1006324 (PMC6114910; doi:10.1371/journal.pcbi.1006324)
Supplement: S1 Text — (PDF) [file pcbi.1006324.s009.pdf]

Equations for body springs

Within the lamprey body, each of the internal links to the centerline points are modeled by Hookean springs and generate equal and opposite forces, tangential to the link, at each node. The magnitude of this restoring force is:

$$F_{int} = |s_{int}(L - L_0)| \tag{1}$$

where s_{int} is the spring constant, L is the evolving length of the spring, and L_0 is the resting length of the spring.

In addition, the links connecting points on the same lateral sides of the body resist extension but not expansion. The magnitude of the force is:

$$F_{skin} = \begin{cases} |s_{skin}(L - L_0)| & L - L_0 > 0 \\ 0 & L - L_0 < 0 \end{cases} \tag{2}$$

where s_{skin} is the spring constant for the lateral springs.
